# HOXC11 drives lung adenocarcinoma progression through transcriptional regulation of SPHK1

**DOI:** 10.1038/s41419-023-05673-8

**Published:** 2023-02-23

**Authors:** Xin Peng, Xiaoli Liu, Wanshan Hu, Yanling Zhou, Lianlian Ouyang, Xintong Peng, Yao Long, Jingyue Sun, Tania Tao, Ling Chen, Ying Shi, Yongguang Tao, Desheng Xiao, Shuang Liu

**Affiliations:** 1 Department of Pathology, Xiangya Hospital, Central South University, Changsha, Hunan 410008 China; 2 Key Laboratory of Carcinogenesis and Cancer Invasion, Ministry of Education, Department of Pathology, Xiangya Hospital, Central South University, Changsha, Hunan 410008 China; 3 NHC Key Laboratory of Carcinogenesis of Ministry of Health (Central South University), Cancer Research Institute; School of Basic Medicine, Central South University, Changsha, Hunan 410008 China; 4 Department of Oncology, Institute of Medical Sciences, National Clinical Research Center for Geriatric Disorders, Xiangya Hospital, Central South University, Changsha, Hunan 410008 China; 5 Department of Medical Genetics, Liuzhou Maternity and Child Healthcare Hospital, Institute of Reproduction and Genetics of Liuzhou City, Affiliated Maternity and Children’s Hospital of Guangxi University of Science and Technology, Liuzhou, Guangxi 545001 China; 6 Department of Dermatology, Hunan Key Laboratory of Medical Epigenomics, The Second Xiangya Hospital, Central South University, Changsha, Hunan 410011 China; 7 Hunan Key Laboratory of Tumor Models and Individualized Medicine; Department of Thoracic Surgery, Second Xiangya Hospital, Central South University, Changsha, Hunan 410011 China

**Keywords:** Non-small-cell lung cancer, Prognostic markers

## Abstract

Lung adenocarcinoma (LUAD) is a fatal threat to human health, while the mechanism remains unclear, and the therapy brings limited therapeutic effects. Transcription factor Homeobox C11 (HOXC11) was previously proved to be related to hind limbs and metanephric development during the embryonic phase, and its role in tumors has been gradually recognized. Our study found that HOXC11 overexpressed in LUAD and was associated with worse overall survival. Moreover, its expression in lung cancer was regulated by IκB kinase α (IKKα), a pivotal kinase in NF-κB signaling, which was related to the ubiquitination of HOXC11. We further proved that HOXC11 could enhance the ability of proliferation, migration, invasion, colony formation, and the progression of the cell cycle in LUAD cells. Meanwhile, it also accelerated the formation of subcutaneous and lung metastases tumors. In contrast, loss of HOXC11 in LUAD cells significantly inhibited these malignant phenotypes. At the same time, HOXC11 regulated the expression of sphingosine kinase 1 (SPHK1) by directly binding to its promoter region. Therefore, we conclude that HOXC11 impacts the development of LUAD and facilitates lung cancer progression by promoting the expression of SPHK1.

## Introduction

With the highest mortality, Lung cancer took approximately 1.796 million people’s lives in 2020 [1]. Lung adenocarcinoma (LUAD) is a major type of lung cancer whose generation is related to driver genes, promoting cancer cell proliferation and tumor development [2]. Targeted therapies have prolonged 64% of LUAD patients’ life who carry the driver mutations [3]. However, on-target or off-target mechanisms related to drug resistance will be a challenge that LUAD patients have to face [4]. In conclusion, more therapy targets need to be explored.

Transcription factor Homeobox C11 (HOXC11) is a member of HOX family, which act as a developmental regulator, expressing in hind limbs [5] and metanephric [6] of mice, co-regulating the joint development with other HOX11 paralogous [7]. Its mutation is related to clubfoot in humans [8]. HOXC11 also has an unignorable effect on tumor progression. It has been reported that HOXC11 is associated with the poor overall survival of colon adenocarcinoma [9], renal clear cell carcinoma [10], and gastric adenocarcinoma [11]. In breast cancer, HOXC11 couples with steroid receptor coactivator 1 to inhibit the expression of differentiation protein CD24 and apoptosis protein PRKC apoptosis WT1 regulator (PAWR) function from promoting the progression of breast cancer [12]. Meanwhile, HOXC11 co-elevates calcium-binding protein S100β with steroid receptor coactivator 1, mediating resistance to endocrine therapy [13]. At the same time, elevated HOXC11 and steroid receptor coactivator 1 expression and recruitments to the S100β promoter region have been observed in malignant melanoma [14]. HOXC11 activates androgen receptors by activating prosaposin, producing more aggressive and endocrine therapy-resistant breast cancer cells when estrogen signaling is blocked [15]. In adult [16] and pediatric [17] acute myelocytic leukemia patients with t(11;12)(p15;q13), a fusion of exon 2 of HOXC11 and exon 12 of NUP98, and chromosomal break of exon 1 of HOXC11 contribute to acute myelocytic leukemia, respectively. In non-small cell lung cancer (NSCLC), It has been reported that HOXC11 knockdown after miR-1197 inhibition can promote cell proliferation and migration in vitro [18]. Apart from this, there is still a gap in reports on the distinct role of HOXC11 in lung cancer, which has aroused our interest in conducting further studies.

NF-κB signaling is almost simultaneously expressed with HOX genes during embryo development, which is essential for the proper development of the embryo and immune function [19]. NF-κB signaling and HOX genes can interact through transcriptional regulation, protein interaction, or related molecules interaction [19]. IκBα, an inhibitory molecule of the NF-κB pathway, is recruited to the regulatory region of HOX genes and inhibits its transcription by directly binding to the N-terminal tails of histone H2A and H4 in skin cells. Therein, IκB kinase α (IKKα) can relieve the transcriptional inhibition of IκBα to HOX genes by reducing IκBα entry into the nucleus, thus promoting the expression of HOX genes [20]. IKKα is a highly conserved protein kinase including protein kinase domain, leucine zipper motif, helix-loop-helix motif, and IKKγ binding domain [21]. In NSCLC, high IKKα expression can promote oncogene activation and NSCLC cells’ proliferation, migration, and tumorigenicity, whose effect is independent of IKKα localization [22].

Sphingosine kinase (SPHK) 1 is a crucial enzyme of sphingolipid metabolism [23], which is activated in the cytoplasm and transported to the cytoplasmic membrane for catalyzing sphingosine 1-phosphate (S1P) production [24]. Besides, SPHK1 was proven to facilitate the occurrence and development of cancer [25]. The high expression of SPHK1 in the lung [26], breast [27], gastric [28], esophageal [29], colon [30], and liver [31] cancer, as well as glioma [32], is closely related to the poor prognosis of patients. SPHK1 acts as an oncogene by promoting tumor cell proliferation [33], migration [34], invasion [35], and chemotherapy resistance [36]. It is also demonstrated that SPHK1 mediates high proliferation, migration, and invasion of NSCLC cells in a STAT3-dependent manner [26] and is of great significance for prognosis prediction of NSCLC [37].

Based on this, we analyzed the expression of HOXC11 in the lung cancer database and explored the effect of HOXC11 on the biological function of LUAD cells. Meanwhile, we also identified a downstream target of HOXC11 and preliminarily verified its function in lung cancer cells. At the same time, we explored the regulatory mechanism of HOXC11 in lung cancer to outline its role of HOXC11.

## Results

### HOXC11 is highly expressed in lung cancer and correlates with poor overall survival of lung adenocarcinoma

**Fig. 1:**
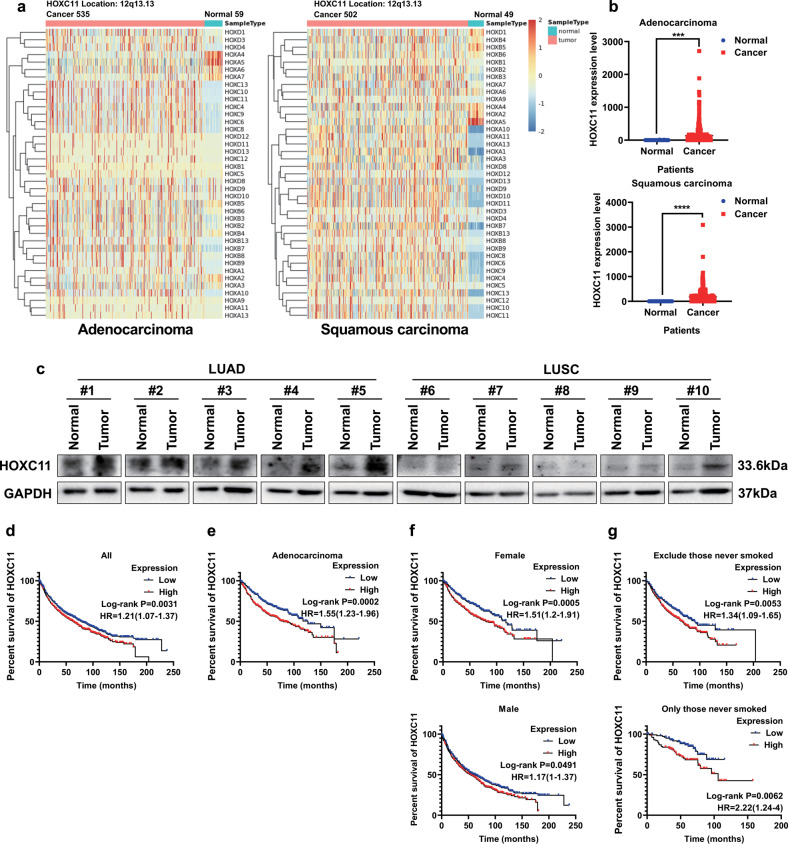
HOXC11 is highly expressed in lung adenocarcinoma and correlates with poor overall survival of LUAD. **a** A heatmap of mRNA level of HOX family genes in LUAD/LUSC and paracancerous samples. **b** mRNA level of HOXC11 in LUAD/LUSC and paracancerous samples. Data in (**a**) and (**b**) are obtained from the TCGA database. **c** HOXC11 protein expression in LUAD/LUSC and paracancerous tissues. **d** The relationship between HOXC11 expression and overall survival of lung cancer patients. **e** The relationship between HOXC11 expression and overall survival of LUAD patients. **f** The relationship between HOXC11 expression and gender of lung cancer patients. **g** The relationship between HOXC11 expression and smoking habits of lung cancer patients. Data from (**d**) to (**g**) come from the Kaplan–Meier Plotter lung cancer dataset. ***P < 0.001, ****P < *0.0001*.

We compared the mRNA level of HOX family in LUAD and lung squamous cell carcinoma (LUSC) samples with paracancerous samples in the TCGA database (Fig. 1a). HOXC11 has a lower mRNA level in paracancerous tissues than in lung cancer tissues, including LUAD or LUSC (Fig. 1b). Clinical LUAD, LUSC, and paired paracancerous tissues were collected to detect the protein expression of HOXC11. The results showed that the HOXC11 protein was highly expressed in LUAD tissues. At the same time, there was no difference between HOXC11 protein expression in LUSC and paracancerous tissues (Fig. 1c), suggesting different expressions or function modes of HOXC11 in LUAD and LUSC. Kaplan-Meier Plotter lung cancer dataset was used to analyze the relationship between mRNA expression of HOXC11 and overall survival (OS) of lung cancer patients (Fig. 1d). LUAD patients with high expression of HOXC11 were found to have shorter OS (Fig. 1e), while there was no significant difference in OS between LUSC patients with high and low HOXC11 expression (Fig. S1a). At the same time, the OS of high HOXC11 level patients was shorter than low-level patients regardless of their gender (Fig. 1f) or smoking habit (Fig. 1g).

### IKKα regulates HOXC11 expression at the post-transcriptional level

**Fig. 2:**
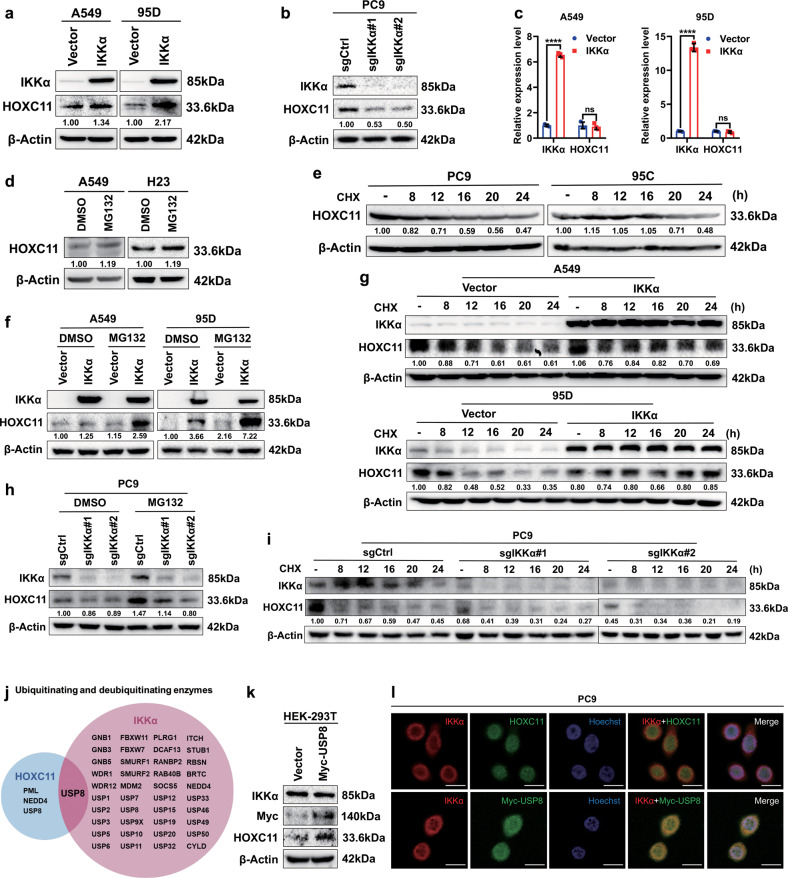
IKKα regulates HOXC11 expression at the post-transcriptional level. **a** Western Blot showed the up-regulation of HOXC11 resulting from IKKα stable overexpression. **b** Western Blot detected the down-regulation of HOXC11 resulting from the IKKα knockout. **c** qPCR analysis of HOXC11 mRNA level after stable overexpression of IKKα. Data are shown as mean ± SD (*n* = 3). **d** Western Blot showing HOXC11 protein cumulation after treatment with MG132 (10 μM, 24 h). **e** Western Blot showed HOXC11 protein degradation after treatment with cycloheximide (10 μg/ml). **f** Western Blot analysis of HOXC11 accumulation after IKKα stable overexpression and MG132 treatment (20 μM, 12 h). **g** Western Blot showed HOXC11 protein expression after IKKα stable overexpression and treatment with cycloheximide (20 μg/ml). **h** Western Blot analysis of HOXC11 accumulation after IKKα knockout and MG132 treatment (20 μM, 10 h). **i** Western Blot showed HOXC11 protein expression after IKKα knockout and treatment with cycloheximide (20 μg/ml). **j** Ubiquitinating and deubiquitinating enzymes of HOXC11 and IKKα predicted by UbiBrowser 2.0. USP8 is shared by HOXC11 and IKKα. **k** USP8 with tag-Myc was instantaneously expressed in HEK293T, and Western Blot detected the protein level of IKKα and HOXC11. **l** Immunofluorescence detected the localization of IKKα (red) relative to HOXC11 (green) or Myc-USP8 (green) in PC9 cells. Scale bars, 20 μm.

To investigate whether HOX gene expression in lung cancer is similarly affected by IKKα as in skin cells described in Introduction, we explored HOXC11 and IKKα expression in NSCLC cells. After establishing IKKα overexpression and knockout cell lines in cells with relatively low and high IKKα expression (Fig. S2a, b), we examined the effects of IKKα overexpression and knockout on HOXC11 protein. We found that its overexpression increased HOXC11 protein levels (Fig. 2a). On the other hand, IKKα knockout significantly reduced the protein expression of HOXC11 (Fig. 2b). Subsequently, we detected the mRNA of HOXC11 in IKKα overexpressed cells. We found that the mRNA level of HOXC11 did not change under IKKα mRNA expression significantly increased (Fig. 2c), proving that IKKα regulated HOXC11 at the post-transcriptional level. Then, we explored one of the most common forms of post-translational modification, ubiquitination, to conduct further research. We selected cells with low HOXC11 expression (Fig. S3b) for further treatment and found an increase in HOXC11 expression after treatment with the proteasome inhibitor MG132 (Fig. 2d). At the same time, the level decreased gradually over time after treatment with Cycloheximide (CHX) in HOXC11 high-expressed cells, which inhibits eukaryotic protein synthesis (Fig. 2e). After that, we performed similar experiments in IKKα overexpression and knockout cell lines. The high expression of IKKα can further increase the protein expression of HOXC11 when MG132 promotes the accumulation of HOXC11 in cells (Fig. 2f), and the decrease of HOXC11 expression caused by CHX can also be recovered by IKKα overexpression (Fig. 2g). The knockout of IKKα impaired the accumulation of HOXC11 caused by MG132 in NSCLC cells (Fig. 2h) and intensified the degradation of HOXC11 raised by CHX (Fig. 2i). These results showed that HOXC11 is affected by the ubiquitin-proteasome pathway. Subsequently, we used UbiBrowser 2.0 and TCGA database to predict ubiquitinating and deubiquitinating enzymes associated with HOXC11 or IKKα and found the expression of ubiquitin-specific protease 8 (USP8) is related to both HOXC11 and IKKα (Figs. 2j, S2c). Accordingly, we overexpressed Myc-labeled USP8 in HEK293T and found that when USP8 expression was elevated, the protein level of HOXC11 increased significantly, but the expression of IKKα was not affected (Fig. 2k). Furthermore, IKKα co-localized with HOXC11 and USP8 in LUAD cells to a certain degree (Fig. 2l). To investigate whether HOXC11 or IKKα has a direct binding to USP8, we used immunoprecipitation to detect the binding situation of USP8 (Fig. S2d). Unfortunately, there was no direct binding of HOXC11 or IKKα to USP8. IKKα overexpression or knockout did not affect the protein levels of USP8 (Fig. S2e, f). However, the knockdown of USP8 in IKKα overexpressed cell line can decrease the protein expression of HOXC11, even though the HOXC11 expression has been upregulated by IKKα (Fig. S2g). At the same time, USP8 transient expression has significantly reduced the level of HOXC11 ubiquitination (Fig. S2h).

### HOXC11 overexpression increases the malignancy of lung cancer cells

**Fig. 3:**
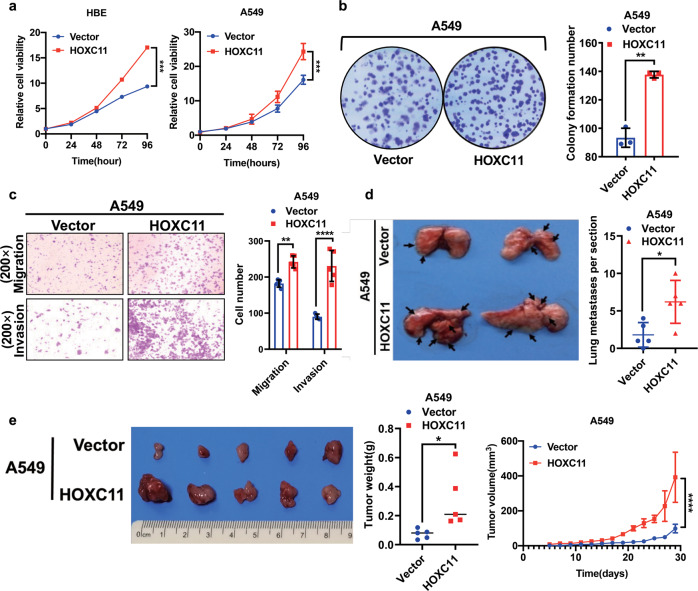
HOXC11 overexpression increases the malignancy of lung cancer cells. **a** Cell counting kit-8 detected the cell viability of HBE and A549 cells after HOXC11 stable overexpression. Data are shown as mean ± SD (*n* = 5). **b** Colony formation assay detected the colony formative ability of HOXC11 overexpression in A549 cells and the quantitative analyses. Data are shown as mean ± SD (*n* = 3). **c** Transwell assay showed the impact of HOXC11 overexpression in migration and invasion ability of A549 cells and the quantitative analyses. Data are shown as mean ± SD (*n* = 5). **d** Lung metastasis of HOXC11 overexpressing A549 cells after intravenous tail injection and the quantitative analyses. Data are shown as mean ± SD (*n* = 5). **e** Subcutaneous xenograft tumors of A549 cells after HOXC11 overexpression. Representative image (left panel), tumor weight (middle panel), and tumor volume (right panel) are shown. Data are shown as mean ± SEM (*n* = 5). *P < 0.05, **P < 0.01, *** P < 0.001, ****P < *0.0001*.

To explore the role of HOXC11 in LUAD, we chose the HOXC11 low-expressed cells (Fig. S3a, b) to establish the HOXC11 stable overexpressed cell lines in LUAD cells (A549 and H23) and normal bronchial epithelial cells (HBE), which have verified by Western Blot and RT-qPCR (Fig. S3c, d). By comparing the proliferation ability of HBE and A549 cells, we found that HOXC11 overexpression increased the proliferating capacity of LUAD cells and the normal bronchial epithelial cell (Fig. 3a). At the same time, overexpression of HOXC11 promoted colony formation (Fig. 3b), migration, and invasion ability (Fig. 3c) and also accelerated the cell cycle progression of LUAD cells (Fig. S3e). Overexpression of HOXC11 also increased the volume and weight of subcutaneous xenograft tumors (Fig. 3e) and the number of lung metastases tumors of LUAD cells (Fig. 3d).

### HOXC11 knockout reduces the malignant features of lung cancer cells

**Fig. 4:**
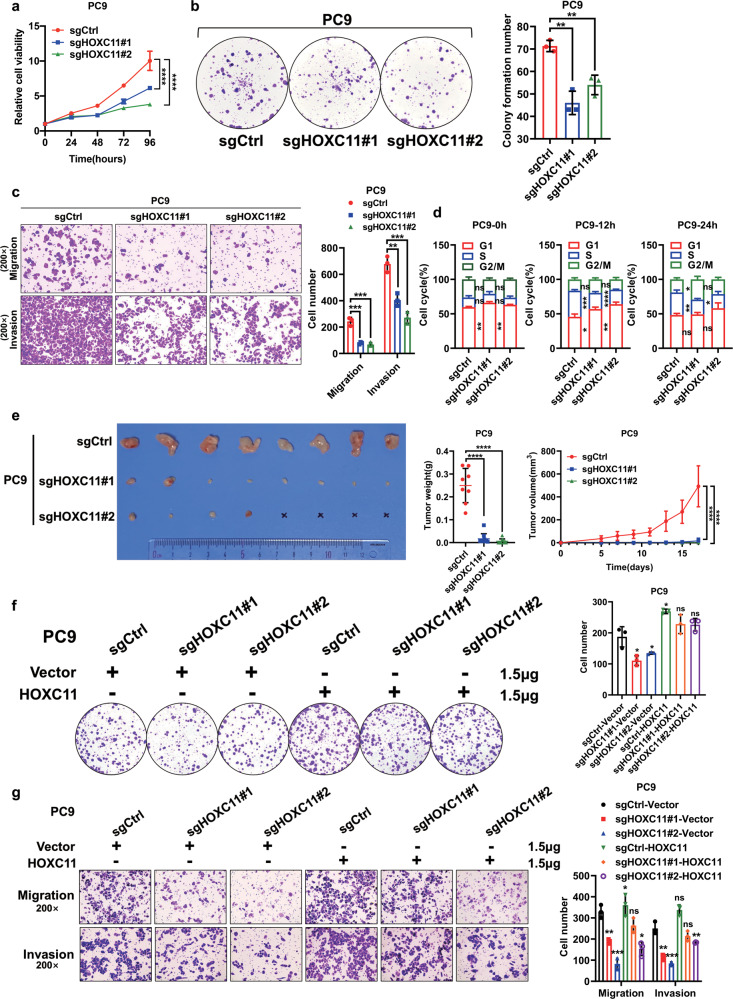
HOXC11 knockout reduces the malignant features of lung cancer cells. **a** HOXC11 knockout decreases the PC9 cells’ proliferation ability detected by cell counting kit-8. Data are shown as mean ± SD (*n* = 5). **b** Colony formation ability of PC9 cells after HOXC11 knockout and the quantitative analyses. Data are shown as mean ± SD (*n* = 3). **c** Transwell assays showed PC9 cell migration and invasion ability of PC9 cells after HOXC11 knockout and the quantitative analyses. Data are shown as mean ± SD (*n* = 3). **d** Flow cytometry detected the cell cycle of PC9 cells after HOXC11 knockout. Differences are compared with the sgCtrl group; data are shown as mean ± SD (*n* = 3). **e** Subcutaneous xenograft tumors formation of HOXC11 knockout cells. Representative image (left panel), tumor weight (middle panel), and tumor volume (right panel) are shown. Data are shown as mean ± SD (*n* = 8). **f** Colony formation showed the colony formative ability of HOXC11 knockout cells after HOXC11 was rescued and the quantitative analyses. Differences were compared with the sgCtrl group transiently transfected with the vector plasmid. Data are shown as mean ± SD (*n* = 3). **g** Transwell assays detected the migration and invasion ability of HOXC11 knockout cells after HOXC11 was rescued by transient transfection and quantitative analyses. Differences were compared with the sgCtrl group transiently transfected with the vector plasmid. Data are shown as mean ± SD (*n* = 3). NS not significant, *P < 0.05, **P < 0.01, *** P < 0.001, ****P < *0.0001*.

To further explore the effect of HOXC11 knockout on LUAD cells, we constructed a HOXC11 knockout cell line (Fig. S4a, b). The proliferation and colony formation ability of PC9 cells were significantly reduced after HOXC11 was knocked out (Fig. 4a, b). The vertical migration and invasion ability of PC9 cells also decreased (Fig. 4c). Flow cytometry shows that HOXC11 knockout cells have a slower cell cycle progression than the control group (Figs. 4d, S4c). Constructing subcutaneous xenograft tumor models and comparing the tumor formative ability found that HOXC11 knockout impaired the ability of tumor formation of PC9 cells (Fig. 4e). Meanwhile, the colony formation, invasion, and metastasis abilities of HOXC11 knockout PC9 cells can be increased considerably after the expression of HOXC11 is rescued (Fig. 4f, g).

### HOXC11 binds to the promoter of SPHK1 to facilitate its expression, predicting a worse prognosis

**Fig. 5:**
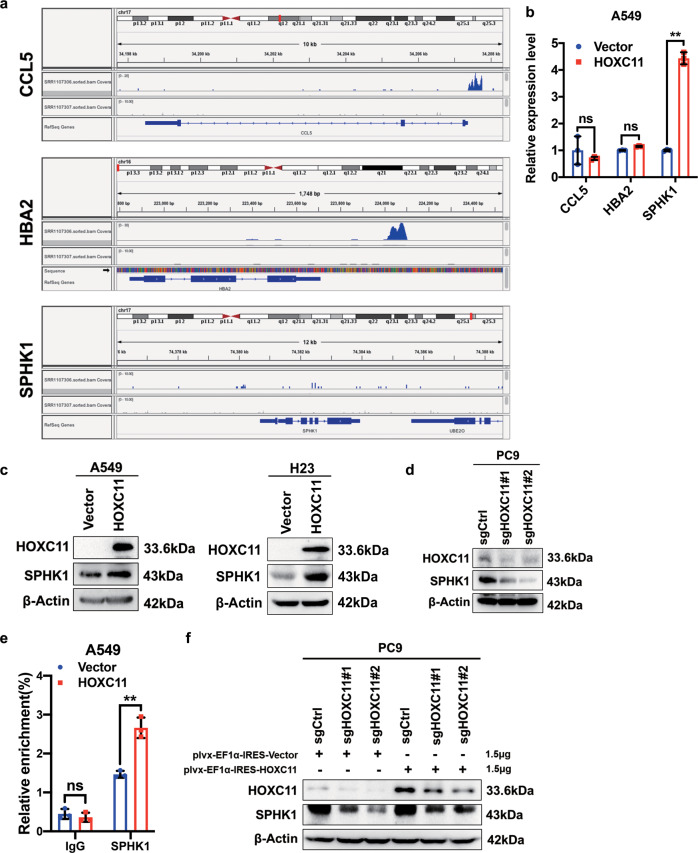
HOXC11 binds to the promoter of SPHK1 to facilitate its expression, predicting a worse prognosis. **a** GSEA analyzed potential binding targets of HOXC11. **b** qPCR analysis of CCL5, HBA2, and SPHK1 expression level after stable overexpression of HOXC11. Data are shown as mean ± SD (*n* = 3). **c** Western Blot analysis of SPHK1 protein expression level after HOXC11 stable overexpression. **d** Western Blot analysis of SPHK1 protein expression level after HOXC11 knockout. **e** Relative enrichment of SPHK1 promoter in HOXC11 stable overexpressing cells. Data are shown as mean ± SD (*n* = 3). **f** Western Blot detected SPHK1 protein expression of HOXC11 knockout cells and that after transiently transfected with 1.5 μg HOXC11 expression plasmid. NS not significant, *P < 0.05, **P < *0.01*.

Due to the tumor-promoted mechanisms of HOXC11 remains unclear, we used GSEA to enrich some HOXC11 expression-related genes, including CCL5, HBA2, and SPHK1 (Fig. 5a), and then detected their mRNA expressive levels in HOXC11 overexpressed cells (Fig. 5b). We found that SPHK1 mRNA was significantly increased after overexpression of HOXC11. To further investigate the effect of HOXC11 on SPHK1 expression, we detected the SPHK1 protein level in HOXC11 overexpressed and knockout cell lines (Fig. 5c, d) and found SPHK1 protein expression parallel correlated with HOXC11 expression. The ChIP assay demonstrated that overexpressing HOXC11 increased the DNA enrichment of the promoter of SPHK1 (Fig. 5e), which indicates that HOXC11 can directly bind to the SPHK1 promoter region to regulate its expression, acting as a transcription factor. Rescuing HOXC11 expression in the HOXC11 knockout cell line can increase SPHK1 expression after reducing SPHK1 induced by HOXC11 knockout (Fig. 5f).

### SPHK1 accelerates the progression of lung cancer

**Fig. 6:**
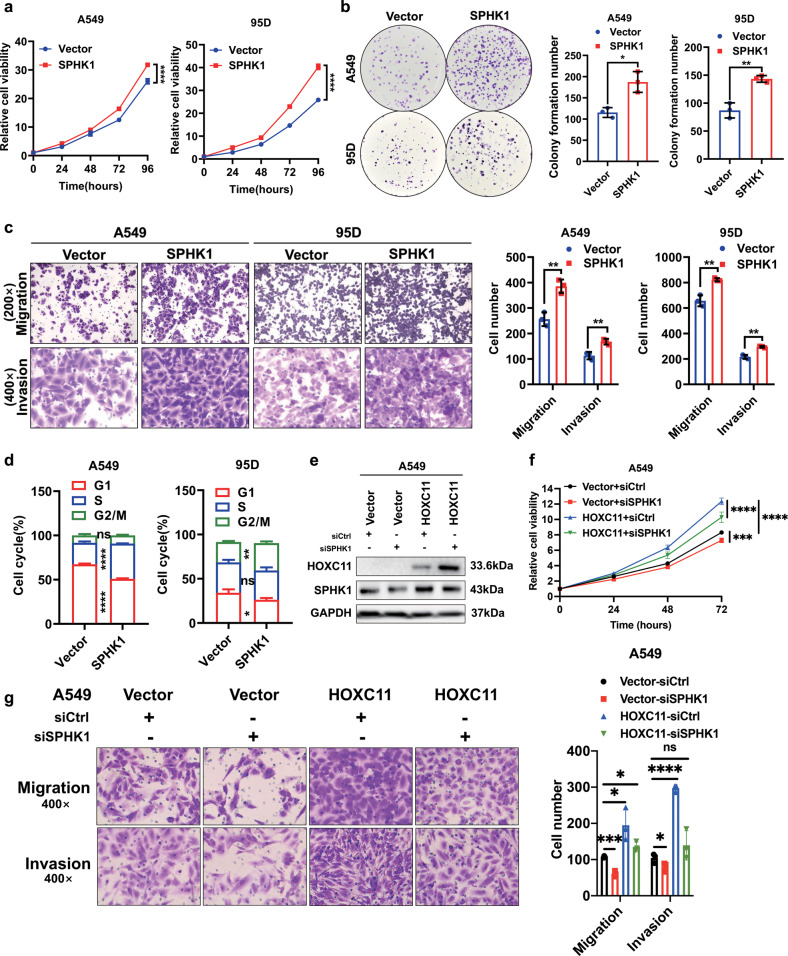
SPHK1 accelerates the progression of lung cancer. **a** Cell proliferation ability of SPHK1 stable overexpressed cells detected by cell counting kit-8. Data are shown as mean ± SD (*n* = 5). **b** Colony formation assays showing the colony formatted ability of SPHK1 stable overexpressed cells and the quantitative analyses. Data are shown as mean ± SD (*n* = 3). **c** Cell migration and invasion ability of SPHK1 stable overexpressed cells detected by Transwell assays and the quantitative analyses. Data are shown as mean ± SD (*n* = 3). **d** Flow cytometry detecting the cell cycle of SPHK1 overexpressed cells. Differences are compared with the sgCtrl group; data are shown as mean ± SD (*n* = 3). **e** Western blot detected the SPHK1 protein level of HOXC11 overexpressing cells with the treatment of SPHK1 interference. **f** Cell counting kit-8 detected the cell viability of HOXC11 overexpressing cell line with the treatment of SPHK1 interference. Data are shown as mean ± SD (*n* = 5). **g** Transwell assays detected the migration and invasion ability of HOXC11 overexpressing cell line with the treatment of SPHK1 interference and the quantitative analyses. Differences were compared to the Vector group transfected with Ctrl siRNA. Data are shown as mean ± SD (*n* = 3). NS not significant, *P < 0.05, **P < 0.01, *** P < 0.001, ****P < *0.0001*.

To validate the function of SPHK1 in lung cancer, we established SPHK1 overexpression cell lines (Fig. S6b) in A549 and 95D cells that low express SHPK1 (Fig. S6a). By detecting the biological functions of SPHK1 overexpressed cell lines, we found that a high level of SPHK1 could promote proliferation (Fig. 6a), migration, and invasion capacity (Fig. 6c) of lung cancer cells. At the same time, they were better at forming clones (Fig. 6b). In addition, the cycle progression of SPHK1 highly expressed cells was also significantly accelerated (Figs. 6d, S6c). Interference with SPHK1 expression in LUAD cells with stable HOXC11 overexpression (Fig. 6e) revealed that accelerated cell proliferation caused by HOXC11 overexpression was slowed down by decreased levels of SPHK1 (Fig. 6f). The downregulation of SPHK1 levels similarly attenuated the migratory and invasive ability of HOXC11 overexpressing cells (Fig. 6g).

### SPHK1 is highly expressed in LUAD and correlates with poor prognosis

**Fig. 7:**
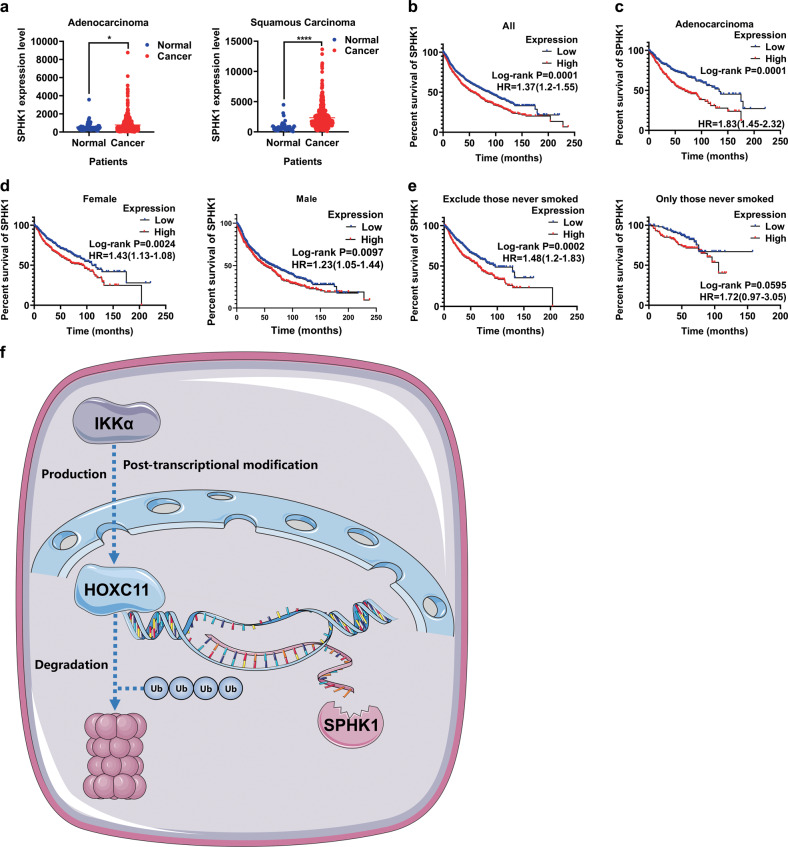
SPHK1 is highly expressed in LUAD and correlates with poor prognosis. **a** mRNA level of SPHK1 in LUAD/LUSC and paracancerous samples in the TCGA database. **b** The relationship between SPHK1 expression and overall survival of lung cancer patients. **c** The relationship between SPHK1 expression and overall survival of LUAD patients. **d** The relationship between SPHK1 expression and gender of lung cancer patients. **e** The relationship between SPHK1 expression and smoking habits of lung cancer patients. Data from (**b**) to (**e**) come from the Kaplan–Meier Plotter lung cancer dataset. **f** A model of HOXC11 bound to the promoter of SPHK1 to regulate its expression and was also affected by IKKα through a ubiquitin-proteasome pathway. *P < 0.05, ****P < *0.0001*.

Then, we focused on lung cancer patients to probe the relationship between SPHK1 expression and prognosis. We analyzed the expression of SPHK1 in LUAD and LUSC from the TCGA database, finding that SPHK1 was highly expressed in both (Fig. 7a). Kaplan-Meier Plotter showed that lung cancer patients with high SPHK1 expression had a shorter OS (Fig. 7b). At the same time, the phenomenon of high SPHK1 expression suggested that a worse prognosis is more pronounced in LUAD (Fig. 7c), which is similar to HOXC11, while there is no significant difference in LUSC (Fig. S7a). In patients with different clinicopathological features, such as smoking habits and gender, the effect of SPHK1 on OS was similar to HOXC11 (Fig. 7d, e).

## Discussion

Lung cancer’s high morbidity and mortality stress families and society. Targeted therapies and immunotherapies have improved the outcome of advanced lung cancer to some extent [38]. However, adequate clinical biomarkers are still insufficient to guide clinical decision-making. In the present study, we identified a new potent prognostic, predictive marker for LUAD. By exploring the expression of HOXC11 in the TCGA database and clinical samples of lung cancer, we found that HOXC11 mRNA levels were upregulated in both LUAD and LUSC. Still, the protein level of HOXC11 was only elevated in LUAD and was closely associated with OS in LUAD patients. This suggests that high expression of HOXC11 in LUAD could be one of the markers of poor prognosis. Subsequently, we validated the function of HOXC11 in LUAD in vitro and in vivo. The high expression of HOXC11 protein significantly enhanced the malignancy of LUAD cells and promoted the proliferation of normal bronchial epithelial cells. On the contrary, the ability of proliferation, migration, invasion, colony formation, and subcutaneous xenograft tumor formation in LUAD cells was significantly decreased, and the cell cycle slowed down after HOXC11 knockout. Furthermore, reverting HOXC11 expression in HOXC11 knockout cells reversed this trend, demonstrating that the HOXC11 protein can promote tumor development in LUAD.

A previous study found that the miR-1197, reversely modulated HOXC11 expression, is highly expressed in NSCLC. Small RNA interference in HOXC11 re-promoted the proliferation and migration of NSCLC cells, which were previously suppressed by miR-1197 Inhibition [18]. These results indicated a different role of HOXC11 in NSCLC. However, these HOXC11-related experiments were based on the miR-1197 that has been inhibited, and there was no direct study of HOXC11 itself. In addition, this study only examined the changes in HOXC11 mRNA level instead of the protein expression. Our results and conclusions are based on studies of HOXC11 protein levels, which are more solid in comparison. We also completed functional and mechanism-related experiments to sketch the contours of HOXC11 in LUAD. Therefore, we are more inclined to believe that HOXC11’s abnormally high expression in LUAD may act as an oncogene.

In the meantime, we were surprised to find that IKKα is an upstream molecule of HOXC11 and can affect its expression at the post-transcriptional level. Ubiquitination-related experiments indicated that one of the potential mechanisms by which IKKα regulates HOXC11 is the ubiquitin-proteasome pathway. Heretofore, researchers have held two different views on the role of IKKα in tumors which need further exploration. It has been reported that targeting IKKα associated with IKKβ can effectively inhibit the development of LUAD, which is caused by simultaneous activation of both canonical and non-canonical NF-κB signaling [39]. However, a study found that loss of IKKα can reduce the survival of KRAS-mutated LUAD patients because that IKKα deletion mutation could up-regulate NOX2, down-regulate NRF2, and promote ROS accumulation [40]. In skin, esophageal, nasopharyngeal squamous cell carcinoma, and pancreatic adenocarcinoma, IKKα can reverse tumor progression driven by its deletion. Conversely, in breast and prostate cancer, IKKα deletion can also attenuate oncogene-induced tumorigenesis and metastasis [41]. In our study, a high level of IKKα facilitated the progression of LUAD by stabilizing HOXC11 from degradation via the ubiquitin-proteasome pathway and upregulating the protein level of HOXC11, suggesting a potential cancer-promoting role for IKKα. Although USP8 could not interact directly with HOXC11, its expression reduced HOXC11 ubiquitination and stabilized its expression while also participating in the post-transcriptional regulation of HOXC11 mediated by IKKα.

In addition, we identified SPHK1 as a downstream target of HOXC11, which directly regulates SPHK1 protein levels by binding to its promoter region. SPHK1 is also highly expressed in lung cancer and is closely associated with poor prognosis in LUAD patients and the malignant phenotype of NSCLC cells. This phenomenon suggested that HOXC11 could impact a series of cellular activities by regulating the expression of SPHK1, thus affecting the progression of LUAD, which may further elucidate the mechanisms of LUAD and develop more alternative therapeutic targets and strategies for patients. Directly reducing HOXC11 synthesis by inhibiting IKKα, promoting HOXC11 degradation through the proteasome, or inhibiting the downstream SPHK-S1P signaling pathway are potential therapeutic targets.

Targeting SPHK1/S1P to modify the therapeutic strategy of tumor patients is a promising approach to enhance the response to immune checkpoint inhibitors in mouse melanoma, breast, and colon cancer models [42]. Fingolimod (FTY720), which can compete with the S1P receptor, has been proven to inhibit the proliferation and promote the apoptosis of colon cancer cells by reducing the mitogenic and growth-promoting signals and lacks toxicity to normal cells [43]. In breast cancer, a high-fat diet and obesity upregulate SPHK1 expression leading to increased S1P. FTY-720 inhibits high-fat diet-induced elevation of lung IL-6 expression and macrophage infiltration, thereby reducing the formation of lung metastases [34]. However, inhibition of S1P affects lymphocyte trafficking, leading to the depletion of circulating lymphocytes, with adverse consequences for immunosuppression [44]. Direct targeting of SPHK1 is also a feasible strategy. In lung cancer, high expression of S1P increases the release of TNF-α and IL-6 from PBMC of lung cancer origin. Compared to the SPHK2 inhibitor Opaganib, which only reduces the release of IL-6 from PBMC of lung cancer origin, inhibition of SPHK1 by PF543 can down-regulate both TNF-α and IL-6 release and more effectively inhibit lung cancer progression [45]. The SPHK1-specific inhibitor SKI-II has also been reported to specifically inhibit the growth of human acute myeloid leukemia cells in vitro while being safe for PBMC from healthy donors. In vivo, SKI-II also inhibited the growth of leukemic xenografts in severe combined immunodeficient mice [46]. In addition, studies have shown that Metformin can downregulate SPHK1 expression, thereby reducing S1P levels and inhibiting the development of ovarian cancer [47]. Compared to other SPHK1 inhibitors, metformin has less toxicity and fewer adverse effects. Several inhibitors of SPHK1 are in development, including SK1-I, DMS, FTY720, Safingol, SK-178, SK-F, etc. [48]. These findings may provide benefits to improve the therapeutic response and clinical outcome of LUAD patients.

However, some things could be improved in this study. Firstly, this study only analyses HOXC11 expression and patient survival in the TCGA database. In contrast, data on HOXC11 expression and patient survival in many clinical samples still need to be included. Secondly, the mechanism of HOXC11 regulation by IKKα needs to be further elucidated. USP8 did not directly bind to either HOXC11 or IKKα, but USP8 reduced the level of ubiquitination of HOXC11, the exact mechanism of which needs to investigate in more detail. Finally, we have not further explored the function, mechanism, and inhibition effects of SPHK1 in vivo. Still, some previous reports indicate the tumor-promoting effects of high SPHK1 expression. Accordingly, we summarize our working model in Fig. 7f. HOXC11 acts as a transcription factor to regulate SPHK1 expression by binding to the promoter region of SPHK1 and promoting the progression of LUAD. In addition, HOXC11 is regulated by IKKα through a post-translational mechanism that may be associated with the ubiquitin-proteasome pathway.

## Materials and methods

### Cell culture and chemicals

Human normal bronchial epithelial cells BEAS-2B and HBE used in this experiment were purchased from ATCC (CRL-9609, CRL-2741, ATCC, VA, USA). Human lung adenocarcinoma cells A549, H358, H1299, and H23 are also from ATCC (CCL-185, CTL-5807, CRL-5803, CRL-5800). Human lung adenocarcinoma cells PC9 and human lung large cell cancer cells 95C and 95D were donated from the cell bank of the Cancer Research Institute of Central South University. The human embryonic renal epithelial cells HEK293T was from ATCC (CRL-11268). BEAS-2B and HEK293T were cultured with DMEM basic medium (C11995500BT, Gibco, NY, USA). HBE, H358, H1299, H23, PC9, A549, 95C, 95D were cultured with RPMI-1640 medium (C11875500BT, Gibco). The culture medium was supplemented with 10% Bovine Calf Serum (B7446, Sigma-Aldrich, MO, USA), penicillin, and streptomycin. All cells were cultured in a 37 °C, 5% CO_2_ incubator, and the medium was changed daily. The CHX (S7418, Selleck, TX, USA) used for cellular experiments concentrations ranged from 10–20 μg/mL, details of which are listed in the legend in Fig. 2. MG132 (S1748, beyotime, China) used in the cell experiments was used at concentrations ranging from 10–20 μM and treatment times of 10–24 h, details of which are also listed in the legend to Fig. 2.

### sgRNA, siRNA, and plasmid transfection

To construct HOXC11, IKKα, and SPHK1 overexpressed cell lines, the pLVX-EF1α-IRES-Puro plasmid (19319, Addgene, Teddington, UK) was used to connect with the sequence of CDS region of these genes. Then, the plasmid was co-transfected with the third-generation plasmid packaging system into HEK293T cells to generate lentivirus and infect cells. Puromycin was used to screen positive clones. For HOXC11 and IKKα knockout, lentiGuide-Puro plasmid (puromycin resistance) (52963, Addgene) was used to carry the sgRNA. The sequence is as follows: IKKα (sg#1-ACGTCTGTCTGTACCAGCAT, sg#2-GTACCAAAAACAGAGAACGA), HOXC11 (sg#1-AATAAGGGCAGCGCTTCTTG, sg#2-TTCCCGAGAAATACTGCAGC). The lentiCas9-Blast (blasticidin resistance) (52962, Addgene) plasmid contains a separate lentiviral construct that delivers hSpCas9, which needs to be used before the lentiGuide-Puro plasmid has been transduced. These two vectors were also transferred into cells by lentivirus. For SPHK1 RNA interference, the human SPHK1-targeting siRNA sequence was referenced from Song et al. [49]. The sequence is as follows: SPHK1 (si-GGCUGAAAUCUCCUUCACGTT). The USP8 expression plasmid contains the CDS sequence of USP8 and is ligated to the pCMV6-Entry plasmid (PS100001, Origene, MD, USA) containing the Myc-DDK tag. For USP8 RNA interference, the si-human-USP8 kit was purchased by Ribobio (siB1151191732-1-5, China).

### Western blot and antibodies

The IP lysate with protease inhibitor was added to cells or tissue fragments for protein extraction. After 2 h of lysis on ice, the supernatant was collected by centrifugation, and the protein concentration was measured by the BCA method. After denaturation, all the proteins were electrophoresed on 80 V for 45 min and then 120 V for 60 min in polyacrylamide gel. Subsequently, the protein was transferred to a polyvinylidene fluoride membrane in ice water. The primary antibody was incubated overnight at 4 °C, and the secondary antibody labeled with horseradish peroxidase was incubated for 2 h at room temperature, followed by chemiluminescence. The anti-HOXC11 mouse antibody was purchased from Novus (1:500, NBP2-00499, Novus, CO, USA), the anti-SPHK1 rabbit antibody, anti-IKKα mouse antibody, Myc-Tag mouse antibody, and anti-GAPDH rabbit antibody were purchased from CST (1:1000, 12071; 1:1000, 11930; 1:1000, 2276; 1:1000, 5174; CST, MA, USA). Anti-β-Actin mouse antibody was purchased from Sigma-Aldrich (1:10000, A1978). Anti-HSP90 mouse antibody was purchased from Proteintech (1:2000, 60318-1-Ig, IL, USA).

### Immunofluorescence

PC9 cells were seeded on glass coverslips and incubated for 48 h. After being fixed with 4% paraformaldehyde, cells were treated with 0.5% Triton X-100, then sealed with 5% goat serum at room temperature for 1 h. The primary antibody was incubated at 4 °C overnight. The anti-HOXC11 mouse antibody was purchased from Novus (1:100, NBP2-00499). The anti-IKKα rabbit antibody and Myc-Tag mouse antibody were purchased from CST (1:3000, 61294; 1:8000, 2276). The fluorescent secondary antibody was diluted with 5% BSA at the ratio of 1:200 and incubated at room temperature for 1 h under dark conditions. The CoraLite 594-conjugated Goat Anti-Rabbit IgG and CoraLite 488-conjugated Affinipure Goat Anti-Mouse IgG were purchased from Proteintech (SA00013-4; SA00013-1). Hoechst stained the nuclei. The photos were caught by laser confocal scanning microscopy (LSM700, ZEISS, Jena, Germany).

### Immunoprecipitation

Cells were collected and an appropriate volume of IP lysate and protease inhibitor was added. After lysing on a shaker for 30 min at 4 °C, centrifuge it to acquire protein lysate. Add the same species of IgG with the primary antibody and the Protein A + G Agarose (P2055, beyotime), and rotate the tubes at 4 °C for 30 min. After the protein concentration was measured, a portion of the protein was taken as an input. 2000μg protein was taken for immunoprecipitation, and 1 μl primary antibody or 1 μl IgG was added. Bind at 4 °C for 1 h, add 20 μl Protein A + G Agarose to the tubes, and rotate at 4 °C overnight. The beads were washed with cold PBS for 5 min and were repeated three times. 40 μg protein was prepared as input. 10 ul suspended beads were taken as IgG or IP group, respectively. The proteins with different molecular weights were separated by polyvinylidene fluoride gel electrophoresis. Then, the protein was transferred to the polyvinylidene fluoride membrane. After incubating the primary and secondary antibodies, the blots were mapped by chemiluminescence.

### Chromatin immunoprecipitation assays

For chromatin immunoprecipitation, formaldehyde (1% final concentration) was added to the 10 cm cell culture dish containing at least 1 × 10^7^ cells with 10 mL medium. The dish was shaken at room temperature for 10 min. Terminate the reaction with 1.25 mol glycine. Remove all the liquid from the dish and add cell lysis buffer to resuspend the cells. Centrifuge the lysate, remove the supernatant, add SDS resolution and protease inhibitor, and lyse on ice for 30 min. Qsonica sonicator was used for ultrasonication (ON 20 s, OFF 20 s, a total of 6 min). The supernatant was retained by 10 min centrifugation at 13000 rpm and kept at 4 °C. Each group contained 300 µg protein for subsequent steps. 2 µg anti-SPHK1 antibody was added to the SPHK1 group, and 2 µL IgG was added to the IgG group. Each group was rotated overnight at 4 °C. Pre-blocked Dynabeads protein G (10004D, Thermo Fisher Scientific, MA, USA) was used to bind the protein-chromatin complex, followed by uncross-linking. The Input group was purified, and quantitative PCR reactions were performed with other groups. Primers used in the experiment were SPHK1 (Forward-AACTTCTTCCTCCGTCTCCG, Reverse-TGTCACTTCTTTGGAGGCCA).

### Total RNA extraction, reverse transcription reaction, and quantitative real-time PCR

More than 1 × 10^6^ cells were collected for total RNA extraction, and TRIzol was added to extract RNA. Chloroform was added to stratify the supernatant, followed by isopropanol to precipitate RNA. After washing the precipitate with 75% ethanol, the RNA was dried at room temperature and dissolved in ribozyme-free water. 1 μg RNA was used to reverse transcript into cDNA after removing genomic DNA by Takara reverse transcription kit (RR047A, Takara, CA, USA). The primer we used were listed below: HOXC11 (Forward- AAAGCCCCAGAGGTTTGTTT, Reverse-AACCTCTGCCCCCAAATAAC), IKKα (Forward- AAGGCCATTCACTATTCTGAGGT, Reverse-GTCGTCCATAGGGGCTCTT), SPHK1 (Forward-GCTGGCAGCTTCCTTGAACCAT, Reverse-GTGTGCAGAGACAGCAGGTTCA), CCL5 (Forward-CCTGCTGCTTTGCCTACATTGC, Reverse-ACACACTTGGCGGTTCTTTCGG), HBA2 (Forward-GACCTGCACGCGCACAAGCTT, Reverse-GCTCACAGAAGCCAGGAACTTG), β-Actin (Forward-CACCATTGGCAATGAGCGGTTC, Reverse-AGGTCTTTGCGGATGTCCACGT). SYBR Green method (B21203, Bimake, TX, USA) was used for real-time fluorescence quantitative PCR (CFX Connect real-time PCR Detection System, bio-rad, DE, USA). The PCR procedure was 95 °C for 10 min, followed by 40 cycles of 95 °C for 2 s, 60 °C for 20 s, and 70 °C for 10 s. β-Actin was used as a reference to obtain genes’ relative expressive level, and the data were analyzed by the 2-ΔΔCt method.

### Cell proliferation, migration, invasion, and plate-colony formation assays

The cell count in suspension was counted under a microscope for cell proliferation assay before planting in 96-well plates. Add 100 μL medium with 1000 cells into the plates. Add 10 μL Cell Counting Kit -8 reagent (B34302, Bimake) at 0 h, 24 h, 48 h, 72 h, and 96 h after the cells were seeded, respectively. The cells were incubated in a 37 °C, 5% CO_2_ environment for 2 h in a dark place. The absorbance value of each well was detected by a microplate reader (Elx800, BioTek, CA, USA). Using 0 h data as a reference, the relative proliferation level was calculated to evaluate cell proliferation ability at each time point.

The Transwell migration assay detected cell migration ability. 5 × 10^4^ cells were suspended in 200 μL 1‰ serum medium and placed in the upper layer of a chamber (353097, Falcon, NY, USA). 600 μL culture medium containing 10% serum was added to the lower layer of the chamber. After 24 h of incubation, the residual cells in the upper layer were removed, fixed with methanol for 15 min, and stained with 0.5% crystal violet for 15 min. The cells in the lower chamber were photographed and counted under the microscope. The migration ability of the cells was evaluated by the number of cells migrating through the chamber.

The Transwell invasion assay detected cell invasion ability. Matrigel (354230, Corning, AZ, USA) was diluted with a serum-free medium at the ratio of 1:10 and added to the upper layer of the chamber. After the diluted Matrigel was dried, 2 × 10^4^ cells were suspended in 200 μL 1‰ serum medium and added to the upper layer of the chamber. A 600 μL medium containing 10% serum was added to the lower layer of the chamber. After being cultured at 37 °C in 5% CO_2_ for 48 h, the Matrigel and remaining cells were removed. Experiencing methanol fixation and 0.5% crystal violet staining, the remaining cell counts were evaluated to assess the invasive ability of cells.

For plate-colony formation assays, 500 cells were planted in six-well plates and cultured in a 5% CO_2_ incubator at 37 °C for 14 days. After 14 d, cells were fixed in methanol for 15 min and stained with 0.5% crystal violet for 15 min. ImageJ was used to acquire the clone count.

### Cell cycle assay and flow cytometry

After cells were cultured in a serum-free medium for 24 h for cell cycle synchronization, cells were cultured in a medium containing 10% serum for 0, 12, or 24 h, respectively. Collect more than 1 × 10^6^ cells, then fix the cell with 70% ethanol and stain it with propidium iodide. RNase was used to remove the RNA from it. Diploid and tetraploid were distinguished by flow cytometry, and FlowJo was used to analyze the cell cycle.

### Subcutaneous xenograft and lung metastatic tumor formation test

All mice receive a standard laboratory diet and are housed under a 12 h light/dark cycle, climate-controlled and pathogen-free conditions. All mice were randomly grouped using the random number table. Collect cells and wash them with PBS solution for the subcutaneous xenograft test. After counting, the cells were diluted to an appropriate concentration and were injected subcutaneously into 5-week-old BALB/c female Nude mice (Hunan SJA Laboratory Animal Co. Ltd, Hunan, China). Each tumor contained 1 × 10^5^ cells with a volume of 50 μL PBS. The tumor volume was measured every 2 days. BALB/c Nude mice were euthanized after transplanted for 29 days and 17 days of A549 (*n* = 5) and PC9 (*n* = 8) cells, respectively. The subcutaneous tumors were stripped and weighed. A metastatic lung tumor was constructed by A549 cells injected via the tail vein (*n* = 5). Each tumor contained 5 × 10^4^ cells with PBS. After 2 months, BALB/c Nude mice were euthanized. The lung tissues were isolated to observe the formation of lung metastases tumors. All procedures for the animal study were approved by the Animal Ethics Committee of the Xiangya Hospital of Central South University and conformed to the legal mandates and federal guidelines. The Ethics Approval ID is 201803416.

### Clinical samples

All lung cancer clinical samples were collected from the Department of Pathology, Xiangya Hospital, Central South University. Patients’ personal information was removed when collecting samples, which met the ethical requirements of protecting patients’ privacy. This study has been approved by the Ethics Committee of Xiangya Hospital of Central South University, and the Ethics Approval ID is 201803415.

### Database massages

The expression data of HOXC11, HOX family genes, and SPHK1 in lung Cancer and adjacent tissues were obtained from The Cancer Genome Atlas (TCGA) database, which contained 535 lung adenocarcinoma tissues and 59 adjacent tissues. Patient survival data were obtained from the Kaplan-Meier Plotter dataset, and the median distinguished the level of mRNA expression. HOXC11 and IKKα ubiquitinating and deubiquitinating enzymes’ prediction came from UbiBrowser 2.0 (http://ubibrowser.bio-it.cn/ubibrowser_v3/). The relevant data between HOXC11 and IKKα and deubiquitinating enzymes’ expression in LUAD was acquired from the TCGA database.

### Statistical methods

The differences between the Control group and another independent sample were compared by unpaired Student’s t-test (two-sided). Cell proliferation data from the last time point were subjected to multiple comparisons using two-way ANOVA. Each group contains at least three samples to ensure statistical analysis. All related data were presented as mean ± SD of three independent experiments performed in triplicate. The mean value and standard deviation were used to describe the deviation distance between each group and the mean. *P* < 0.05 was considered to have a statistical difference. Graph Pad Prism software version 9.0 was used for all statistical analyses.

## Supplementary information

Supplementary figure 1

Supplementary figure 2

Supplementary figure 3

Supplementary figure 4

Supplementary figure 6

Supplementary figure 7

Supplementary figure legend

checklist

Original Data File

## Data Availability

The datasets we used in this paper are public datasets, including The Cancer Genome Atlas database (https://portal.gdc.cancer.gov/), Kaplan–Meier Plotter dataset (https://kmplot.com/analysis/index.php), and UbiBrowser 2.0 (http://ubibrowser.bio-it.cn/ubibrowser_v3/).
