# Supplementary figures and images for: HOXC11 drives lung adenocarcinoma progression through transcriptional regulation of SPHK1
Source: Cell Death Dis. 2023 Feb 23;14(2):153. doi: 10.1038/s41419-023-05673-8 (PMC9950477; doi:10.1038/s41419-023-05673-8)

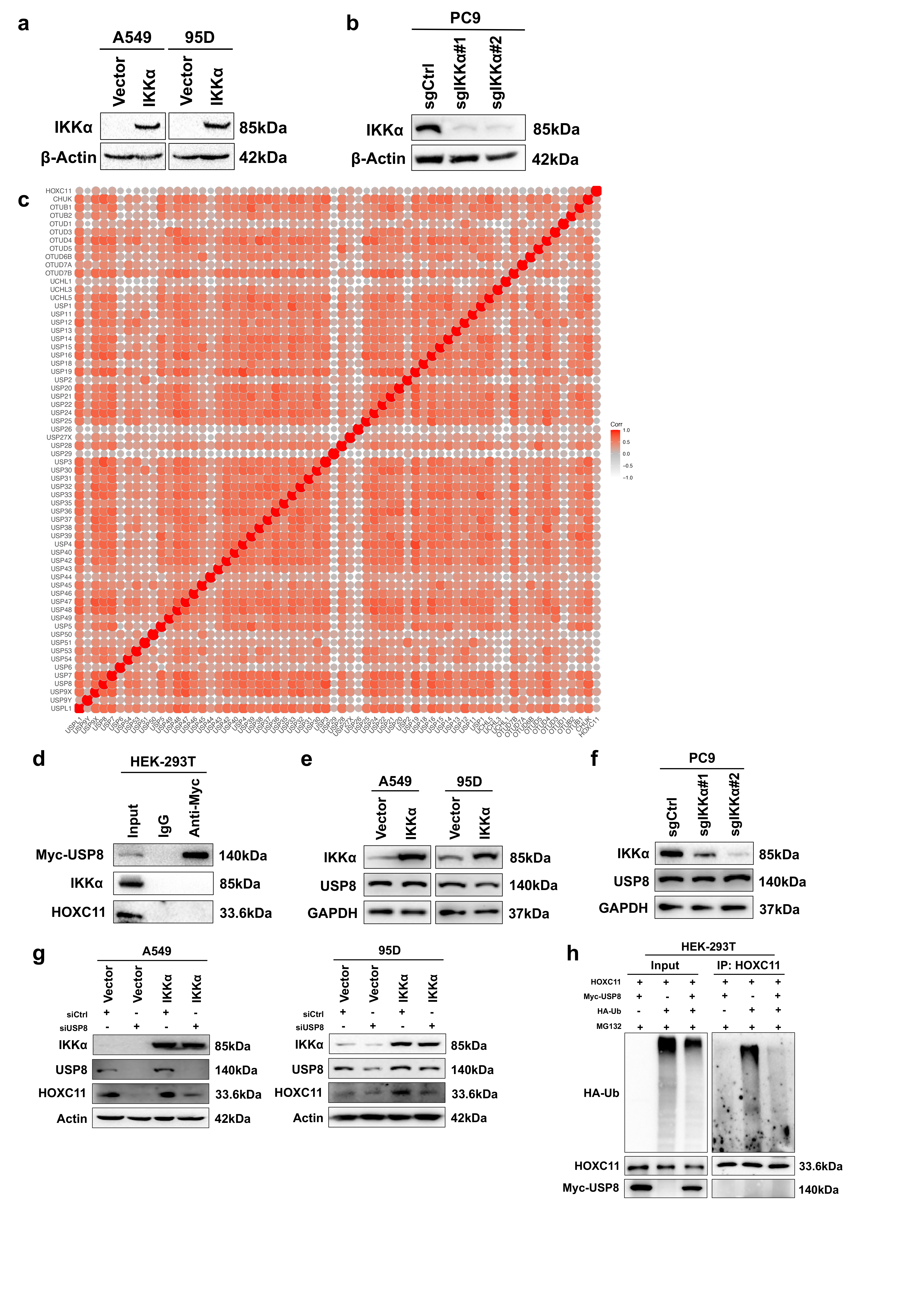

Supplement: Supplementary file 2 — Supplementary figure 2 [file 41419_2023_5673_MOESM2_ESM.tif]
