# Supplementary material for: HOXC11 drives lung adenocarcinoma progression through transcriptional regulation of SPHK1
Source: Cell Death Dis. 2023 Feb 23;14(2):153. doi: 10.1038/s41419-023-05673-8 (PMC9950477; doi:10.1038/s41419-023-05673-8)
Supplement: Supplementary file 7 — Supplementary figure legend [file 41419_2023_5673_MOESM7_ESM.docx]

**Supplementary Material**

**Figure legends:**

**Supplementary Figure 1. Related to Figure 1. (a)** The relationship between HOXC11 expression and overall survival of LUSC patients.

**Supplementary Figure 2. Related to Figure 2. (a)** Western blot showing the successful overexpression of IKKα. **(b)** Western blot showed the knockout of IKKα. **(c)** The consistency of HOXC11 or IKKα mRNA expression and Ubiquitinating or deubiquitinating enzymes is predicted by the TCGA database. **(d)** Exogenous Myc-USP8 and HOXC11 or IKKα were transiently transfected in HEK293T cells. The USP8 was immunoprecipitated by the Myc-tag antibody. IgG served as a negative control. HOXC11 and IKKα were detected by Western blot. **(e)** Western blot showed the USP8 expression after IKKα overexpression. **(f)** Western blot showed the USP8 expression after IKKα knockout. **(g)** The HOXC11 protein level was detected by Western blot after the small interfering RNA of USP8 was transferred in IKKα overexpressed cell or not. **(h)** The lysates of HEK-293T cells containing 800μg of total protein were immunoprecipitated with an anti-HOXC11 antibody, and ubiquitination was examined using an anti-HA antibody.

**Supplementary Figure 3. Related to Figure 3. (a)** qPCR analysis of HOXC11 mRNA expression in normal bronchial epithelial cells or diverse lung cancer cells. **(b)** Western blot showing the HOXC11 protein expression in normal bronchial epithelial cells or lung cancer cells. **(c)** Western blot showed the successful overexpression of HOXC11 in normal bronchial epithelial cells and LUAD cells. **(d)** qPCR detecting HOXC11 mRNA level after that stable overexpression. **(e)** Flow cytometry detected the cell cycle after HOXC11 overexpression. **** P<0.001, ****P<0.0001*

**Supplementary Figure 4. Related to Figure 4. (a)** Western blot showed the successful knockout of HOXC11. **(b)** qPCR analysis of HOXC11 mRNA expression in HOXC11 knockout cells. **(c)** The supplementary figure of figure 4(d) is the cell cycle of PC9 cells after HOXC11 knockout. *ns, not significant, *P<0.05, **P<0.01, *** P<0.001, ****P<0.0001*

**Supplementary Figure 6. Related to Figure 6. (a)** Western blot showed the SPHK1 protein expression in normal bronchial epithelial cells or lung cancer cells. **(b)** Western blot showed the successful overexpression of SPHK1 in lung cancer cells. **(c)** The supplementary figure of figure 6(d) shows the cell cycle of A549 and 95D cells after SPHK1 overexpression. *ns, not significant, *P<0.05, **P<0.01, ****P<0.0001*

**Supplementary Figure 7. Related to Figure 7**

**(a)** The relationship between SPHK1 expression and overall survival of LUSC patients.
