# Supplementary material for: HOXC11 drives lung adenocarcinoma progression through transcriptional regulation of SPHK1
Source: Cell Death Dis. 2023 Feb 23;14(2):153. doi: 10.1038/s41419-023-05673-8 (PMC9950477; doi:10.1038/s41419-023-05673-8)

Uncropped whole blots of figure 1 used in this study.

Corresponds to Figure 1c, the first to the fifth column.

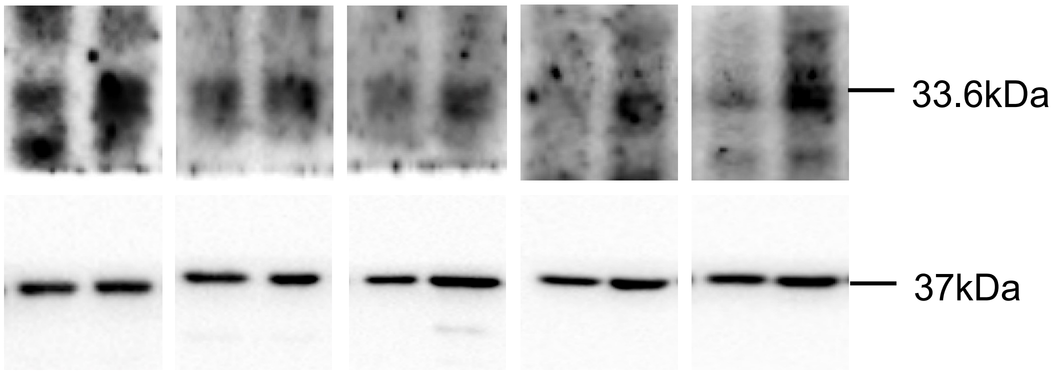

Corresponds to Figure 1c, the sixth to the tenth column.

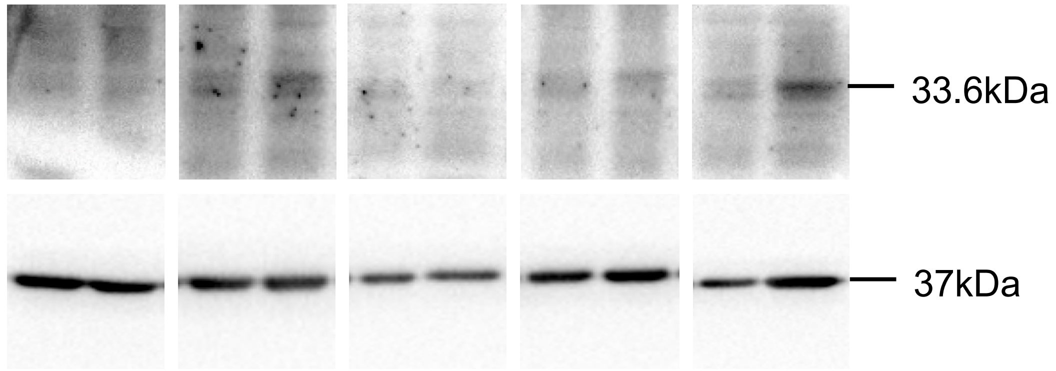

Uncropped whole blots of figure 2 used in this study.

Corresponds to Figure 2a, the first and the second column.

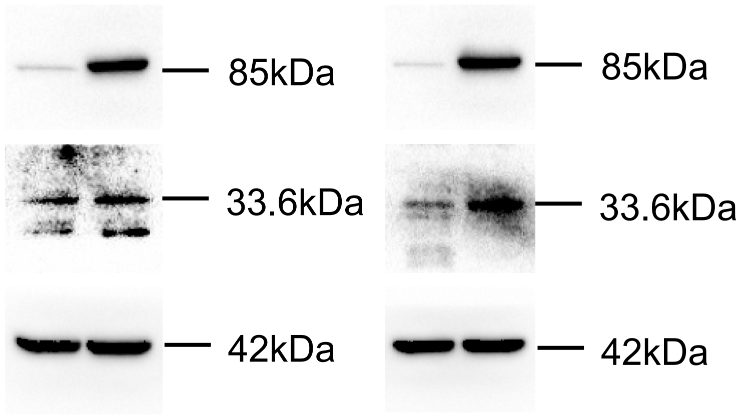

Corresponds to Figure 2b.

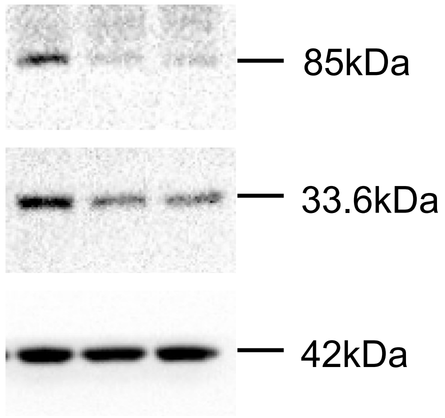

Corresponds to Figure 2d, the first and the second column.

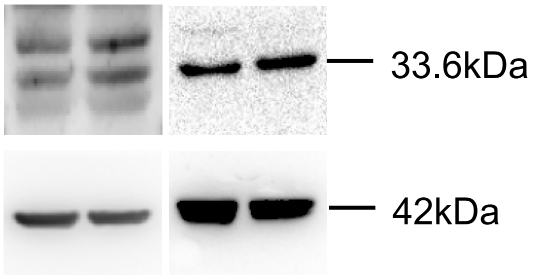

Corresponds to Figure 2e, the first and the second column.

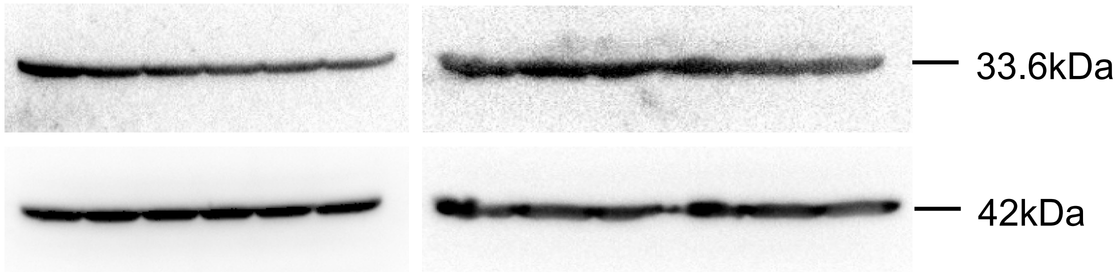

Uncropped whole blots of figure 2 used in this study.

Corresponds to Figure 2f, the first and the second column.

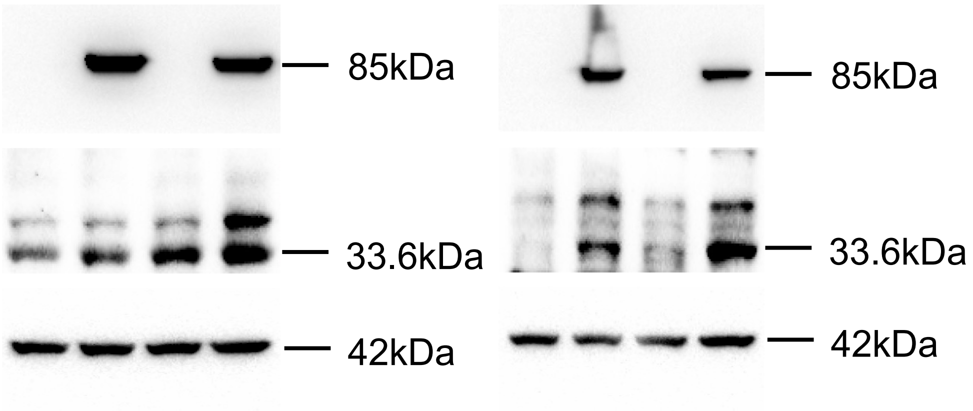

Corresponds to Figure 2g, the first column.

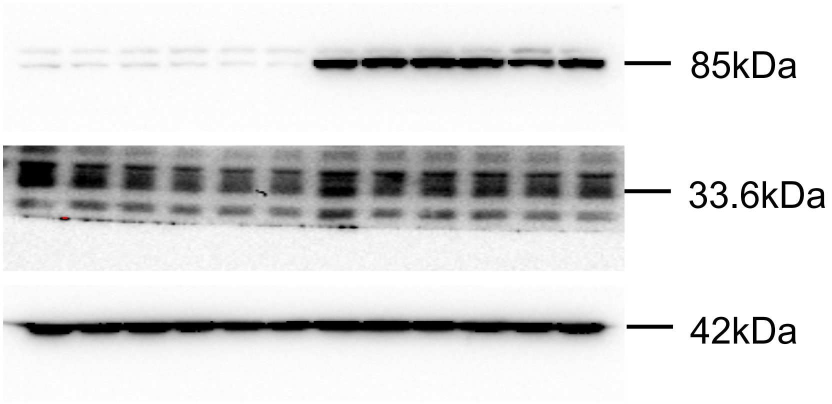

Corresponds to Figure 2h.

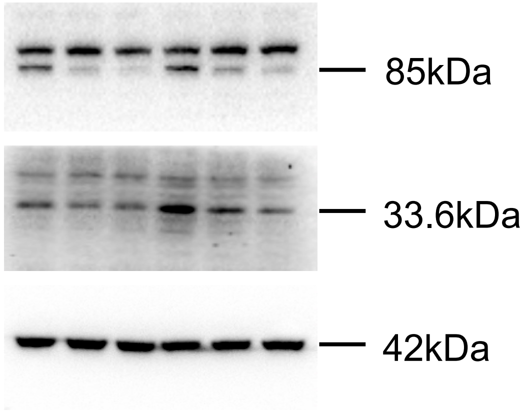

Corresponds to Figure 2g, the second column.

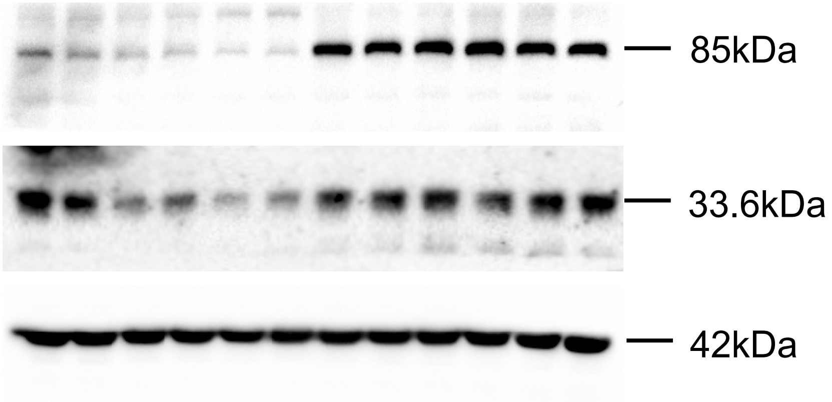

Corresponds to Figure 2i, the first and the second column.

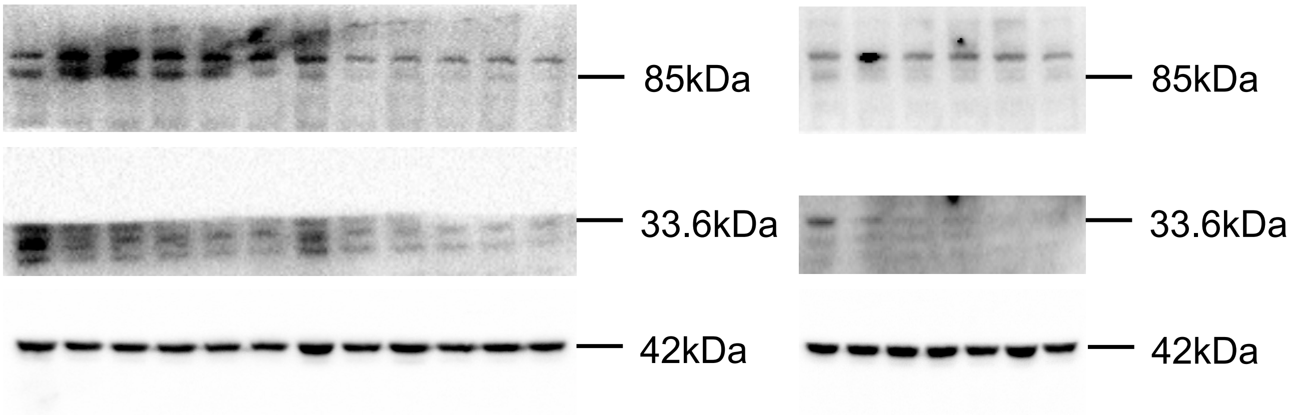

Uncropped whole blots of figure 2 used in this study.

Corresponds to Figure 2k.

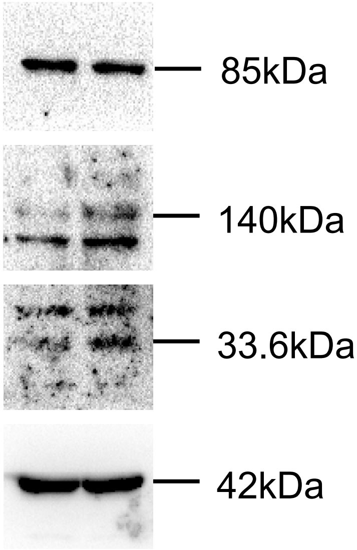

Uncropped whole blots of figure 5 used in this study.

Corresponds to Figure 5c, the first and the second column.

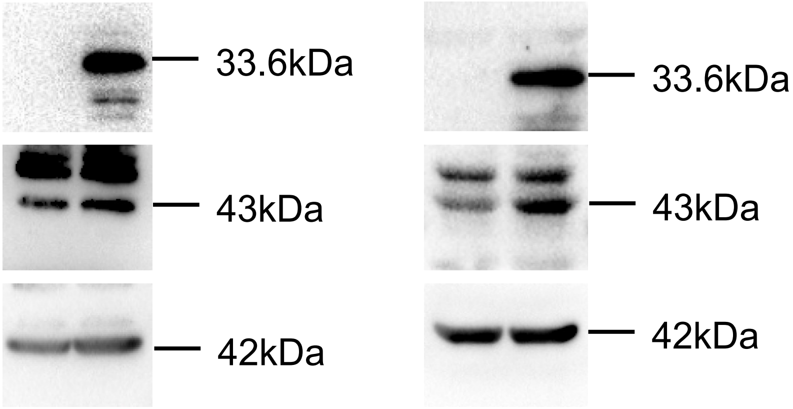

Corresponds to Figure 5d.

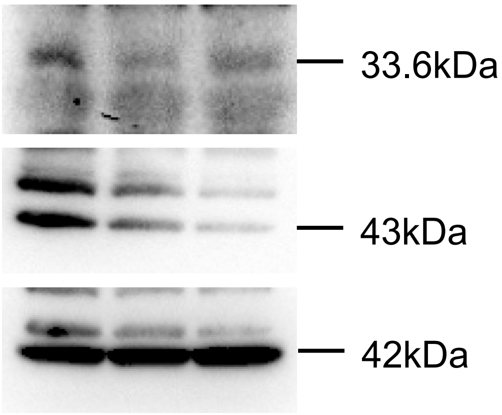

Corresponds to Figure 5f.

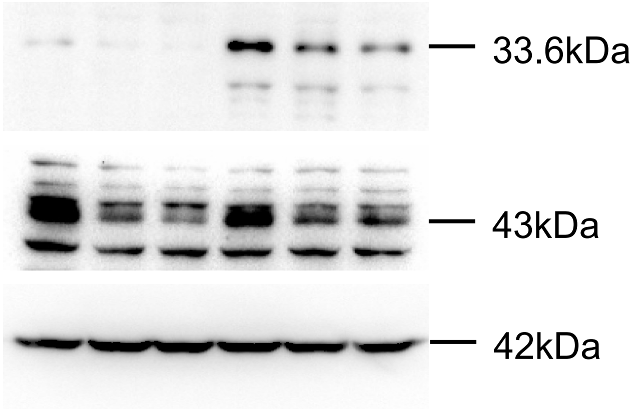

Uncropped whole blots of figure 6 used in this study.

Corresponds to Figure 6e.

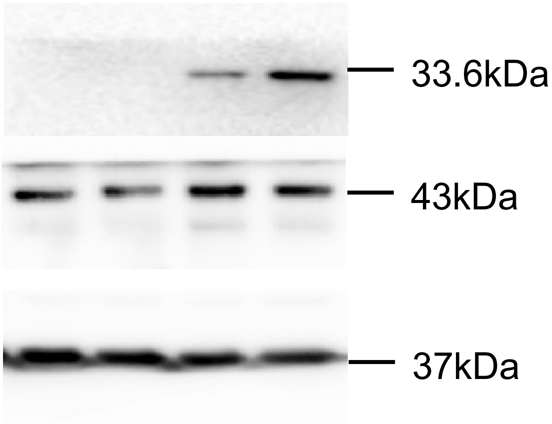

Uncropped whole blots of figure S2 used in this study.

Corresponds to Figure S2a, the first and the second column.

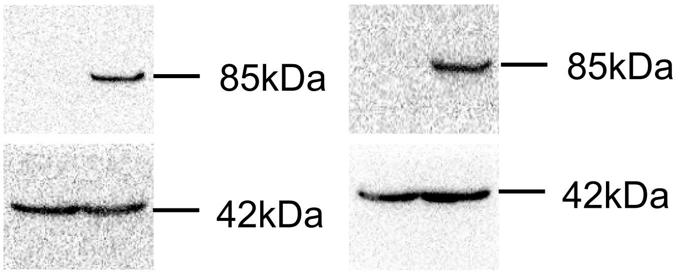

Corresponds to Figure S2d.

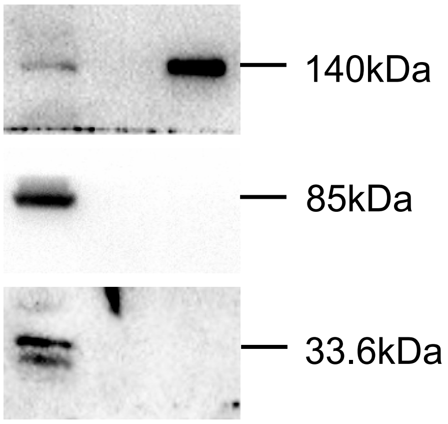

Corresponds to Figure S2b.

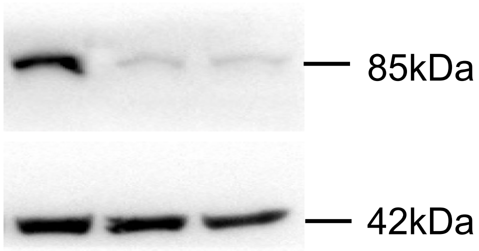

Corresponds to Figure S2e, the first and the second column.

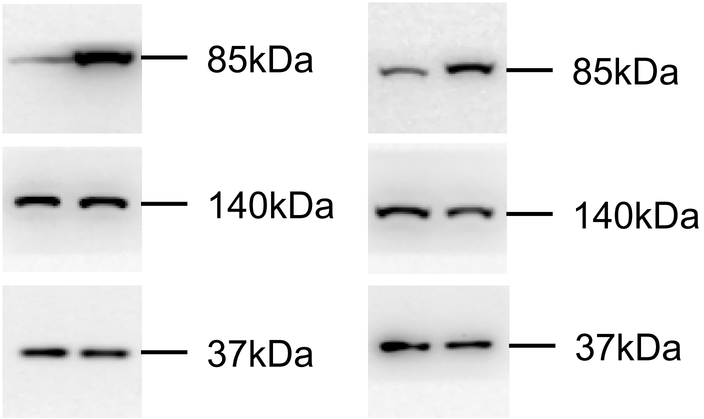

Corresponds to Figure S2f.

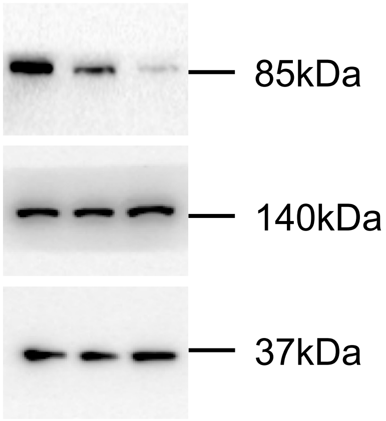

Corresponds to Figure S2g, the first and the second column.

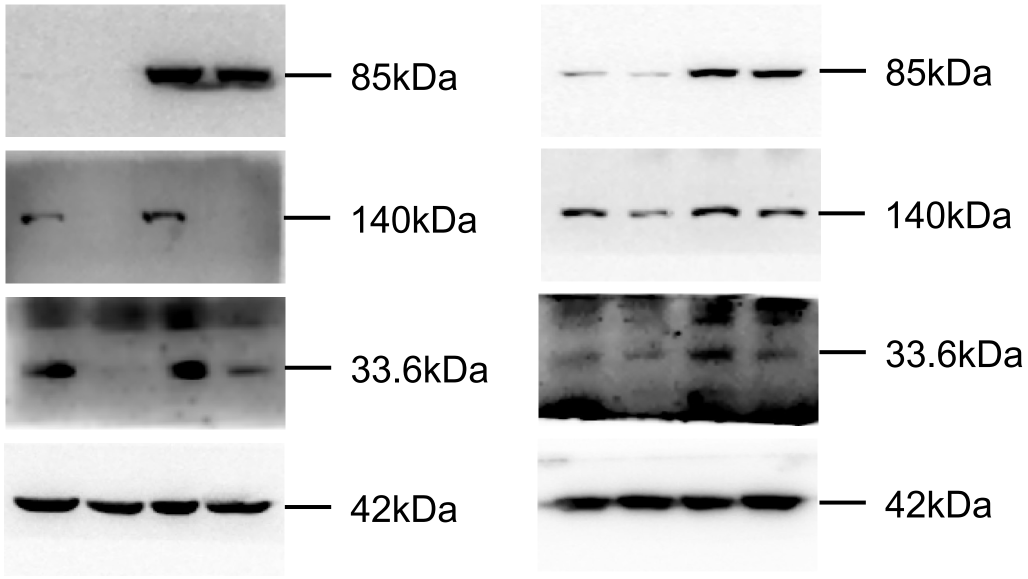

Uncropped whole blots of figure S2 used in this study.

Corresponds to Figure S2h, the first and the second column.

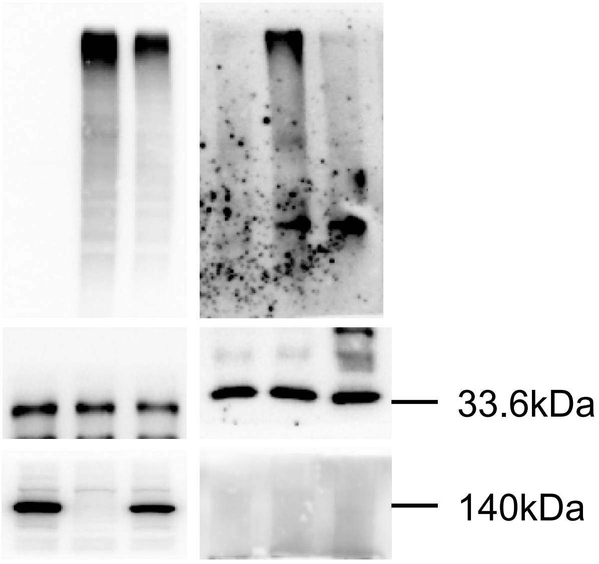

Uncropped whole blots of figure S3 used in this study.

Corresponds to Figure S3b.

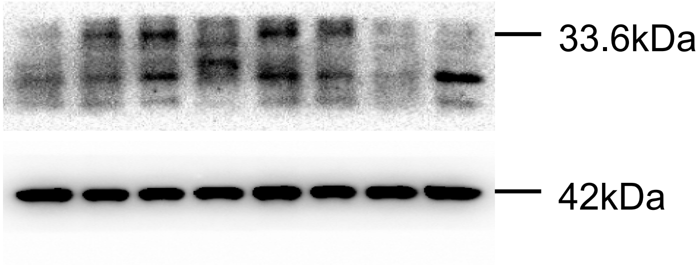

Corresponds to Figure S3c, the first to the third column.

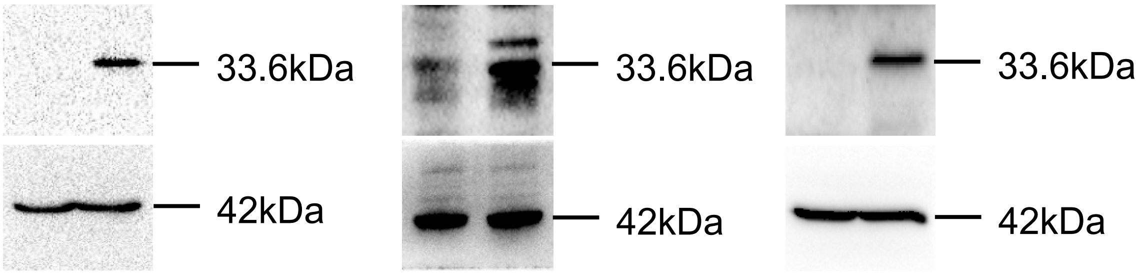

Uncropped whole blots of figure S4 used in this study.

Corresponds to Figure S4a.

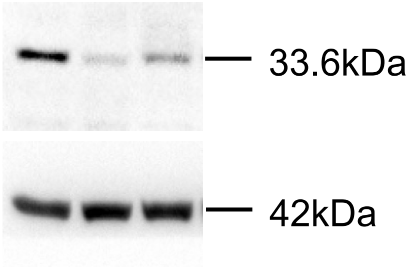

Uncropped whole blots of figure S6 used in this study.

Corresponds to Figure S6a.

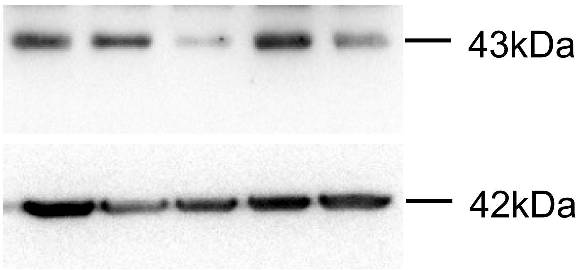

Corresponds to Figure S6b, the first and the second column.

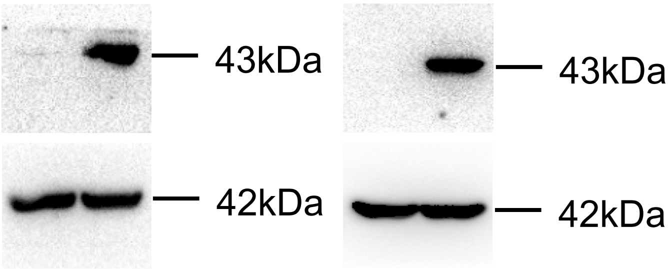

Supplement: Supplementary file 9 — Original Data File [file 41419_2023_5673_MOESM9_ESM.pdf]
